# Sphincter-saving surgery versus abdominoperineal resection in low rectal cancer: the role of indocyanine green fluorescence angiography in surgical decision-making

**DOI:** 10.3389/fonc.2026.1873934

**Published:** 2026-07-09

**Authors:** Mihaela C. Misca, Sorin V. Petrea, Roxana D. Boanta, Sorin Aldoescu, Eduard Catrina, Mihaela E. Vilcu, V. Grigorean, V. Strambu, Iulian Brezean

**Affiliations:** 1 Dr. I. Cantacuzino Clinical Hospital and Carol Davila University of Medicine and Pharmacy, Bucharest, Romania; 2 Parhon Endocrinology Institute, Bucharest, Romania; 3 Bagdasar-Arseni Emergency Clinica Hospital Bucharest and Carol Davila University of Medicine and Pharmacy Bucharest, Bucharest, Romania; 4 Dr. Carol Davila Nephrology Hospital Bucharest Romania and Carol Davila University of Medicine and Pharmacy Bucharest, Bucharest, Romania

**Keywords:** abdominoperineal resection, coloanal anastomosis, fluorescence angiography, indocyanine green, intersphincteric resection, low rectal cancer, pull-through, sphincter-saving surgery

## Abstract

**Background:**

The choice between sphincter-saving surgery and abdominoperineal resection (APR) for low rectal cancer balances oncological radicality against functional preservation. Indocyanine green fluorescence angiography (ICG-FA) provides objective intraoperative assessment of bowel perfusion at the colonic stump, where ischemia is a leading cause of anastomotic failure.

**Materials and methods:**

In this prospective, single-center, descriptive cohort study (January 2021–December 2025) of more than 400 patients operated for colorectal cancer, 70 had low rectal cancer (≤6 cm from the anal verge) and 27 underwent sphincter-saving surgery — 7 intersphincteric resections, 4 immediate low colorectal or coloanal anastomoses, and 16 two-staged Turnbull–Cutait pull-through procedures. ICG-FA of the colonic stump was performed in 22 patients (81.5%), with proximal repositioning of the resection line whenever fluorescence was inadequate.

**Results:**

ICG-FA prompted modification of the resection line in 9 of 22 patients (40%), almost always proximally, but never altered the decision between sphincter preservation and APR. A radical (R0) resection was achieved in 19 of 21 patients with margin status recorded; two had an involved circumferential margin (R1), both in locally advanced node-positive tumors. Postoperative complications occurred in 15 of 27 patients (56%), mostly Clavien–Dindo grade I–II. There was no 30-day mortality; one late in-hospital death from an independent myocardial infarction occurred beyond 30 days.

**Conclusions:**

Sphincter-saving surgery for low rectal cancer is feasible but carries substantial morbidity. ICG-FA is a reproducible, low-risk adjunct that informs intraoperative decision-making; whether it reduces anastomotic complications requires confirmation in randomized studies.

## Introduction

Rectal cancer remains one of the most prevalent malignancies worldwide and represents a major source of morbidity and mortality. Within this entity, low rectal cancer — conventionally defined as a tumor whose lower margin lies within 6 cm of the anal verge — continues to confront the surgeon with one of the most delicate trade-offs in oncologic surgery: the balance between radicality, anal sphincter preservation, and acceptable functional outcome.

For decades, abdominoperineal resection (APR) with permanent end colostomy was considered the standard treatment for tumors of the distal rectum. The advent of total mesorectal excision (TME), the refinement of stapling devices and the systematic use of neoadjuvant chemoradiotherapy have progressively pushed the lower limit of safe sphincter preservation further down the anal canal. As a result, low anterior resection with coloanal anastomosis, intersphincteric resection (ISR), and the pull-through (Turnbull–Cutait) technique have become valid alternatives for selected patients, allowing avoidance of a permanent stoma without compromising oncological outcomes.

Despite this evolution, choosing between sphincter-saving surgery and APR is rarely straightforward. The surgeon must integrate tumor-related variables (distance to the anal verge, response to neoadjuvant therapy, threatened circumferential resection margin, sphincter infiltration), patient-related variables (age, comorbidities, baseline continence, body habitus, narrow male pelvis) and technical considerations linked to the planned anastomosis. Several studies have shown that, although global quality-of-life scores are comparable between APR and sphincter-preserving procedures, the latter offer a measurable advantage in terms of body image, sexual and urinary function, and emotional adjustment, particularly in younger patients (1, 2). Conversely, low coloanal and intersphincteric anastomoses are associated with significant rates of anastomotic leak, low anterior resection syndrome, and stoma-related complications, which can substantially erode the expected functional benefit (3, 4).

This decision has become even more nuanced under the contemporary multimodal treatment paradigm. Long-course chemoradiotherapy and total neoadjuvant therapy can produce significant tumor downsizing and downstaging, occasionally converting a tumor that initially appeared unsuitable for sphincter preservation into one amenable to a restorative procedure. The increasing acceptance of organ preservation strategies in clinically complete responders further blurs the historical boundaries of surgical indications. Within this evolving framework, the surgeon facing a tumor in the lowest rectum must combine oncological judgement, anatomical assessment by high-resolution magnetic resonance imaging, response to neoadjuvant therapy, and a realistic estimate of the achievable functional outcome before committing to a definitive operative plan.

Among the modifiable risk factors for anastomotic failure in low rectal surgery, inadequate perfusion of the proximal colonic stump is recognized as a leading mechanism. The historical reliance on subjective intraoperative criteria — color of the bowel, palpable mesenteric pulse, active bleeding from the cut edge — has well-documented limitations, especially after high ligation of the inferior mesenteric artery and extensive splenic flexure mobilization (5). Indocyanine green fluorescence angiography (ICG-FA) has been introduced into colorectal practice precisely to overcome this limitation. After intravenous administration, ICG binds rapidly to plasma proteins and emits a near-infrared signal that allows real-time visualization of bowel perfusion. Multiple meta-analyses and the recent IntAct multicenter randomized trial have suggested that intraoperative ICG-FA reduces the incidence of anastomotic leak after rectal resection and frequently leads to a change in the planned resection line (6–9).

On this background, our team has adopted, since 2021, a structured intraoperative use of ICG-FA in all sphincter-saving procedures performed for low rectal cancer. The aim of the present study was twofold: first, to describe our institutional experience over five consecutive years in selecting between sphincter-saving surgery and APR for low rectal tumors; and second, to evaluate the actual influence of ICG perfusion assessment on intraoperative decision-making and on the postoperative course, with particular attention to perfusion-related complications. The study is descriptive and was neither designed nor powered to establish a causal effect of ICG-FA on outcomes.

## Materials and methods

### Study design and population

This is a prospective, observational, single-center, descriptive cohort study carried out in a tertiary academic surgical department between January 2021 and December 2025. All consecutive patients operated for histologically proven colorectal adenocarcinoma during the study period were entered into a dedicated electronic database. The protocol was approved by the local institutional ethics committee, and all patients signed informed consent for surgery, photographic documentation, prospective follow-up, and anonymous use of clinical data for research purposes.

**Figure 1:**
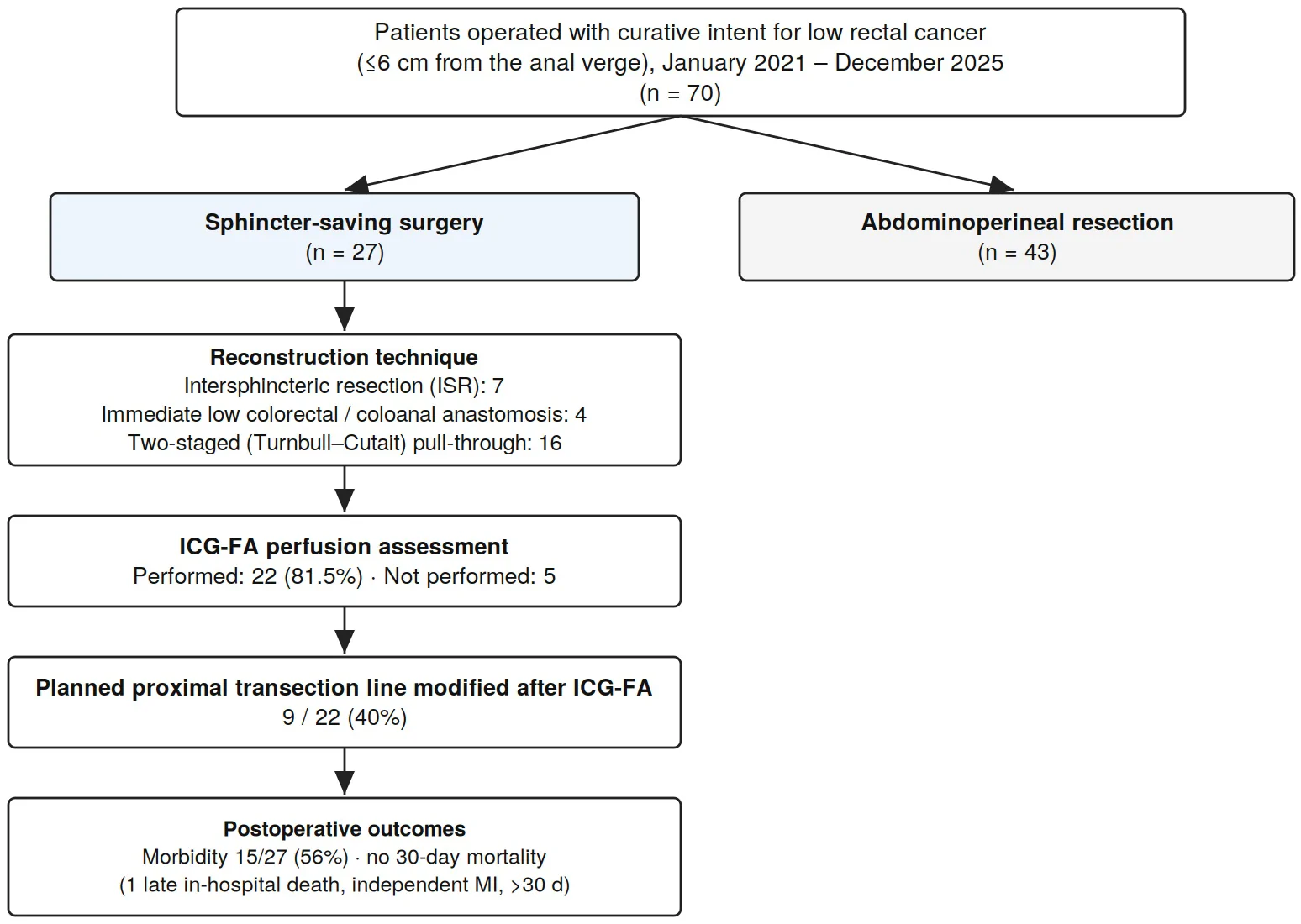
Cohort flow diagram showing patient selection and surgical allocation between January 2021 and December 2025.

Out of more than 400 patients with colorectal cancer included in the global cohort, 70 patients (17.4%) presented with low rectal cancer, defined as adenocarcinoma whose distal edge was located within 6 cm of the anal verge on rigid proctoscopy and pelvic magnetic resonance imaging. These 70 patients constitute the main subgroup analyzed in the present report. The flow of the study cohort is summarized in **Figure 1**.

Pre-treatment staging included colonoscopy with biopsy, contrast-enhanced thoraco-abdominal computed tomography, high-resolution pelvic magnetic resonance imaging, and serum carcinoembryonic antigen measurement. Patients with locally advanced disease — cT3 with threatened circumferential resection margin, cT4, or node-positive tumors — received neoadjuvant therapy according to the multidisciplinary tumor board recommendation, in the form of either long-course chemoradiotherapy (50.4 Gy with concurrent fluoropyrimidine) or short-course radiotherapy (5 × 5 Gy) followed by delayed surgery. Restaging pelvic MRI was performed eight to ten weeks after the completion of neoadjuvant treatment to assess tumor regression, the integrity of the mesorectal fascia, and to confirm the indication for sphincter-saving surgery whenever feasible.

Inclusion criteria for sphincter-saving surgery were: histologically confirmed rectal neoplasia; tumor classified cT1–cT3, any N, M0 after neoadjuvant treatment when indicated; preserved baseline sphincter function (clinical assessment supported, when needed, by anorectal manometry); absence of macroscopic involvement of the external sphincter or the levator ani; and patient preference for sphincter preservation after detailed counselling. Patients with bulky T4 tumors infiltrating the sphincter complex, severe pre-existing fecal incontinence, or general unfitness for prolonged pelvic surgery were directed towards APR. The cohort reflects consecutive practice and therefore includes early lesions — most notably one laterally spreading rectal tumor with high-grade dysplasia managed without neoadjuvant therapy; these are reported transparently and are identified in **Table 1**.

**Table 1:** Baseline oncological and clinicopathological characteristics (n = 27).

Variable	Value
Age, median (range), years	65 (36–85)
Sex, male/female, n	16/11
Tumor distance from anal verge, median (range), cm	5 (1–7)
Neoadjuvant therapy, n (%)	26 (96); 1 untreated (laterally spreading tumor)
Clinical cT stage (recorded in 16), n	cT1 1; cT2 5; cT3 7; cT4 3
Clinical cN stage (recorded in 16), n	cN0 4; cN1 4; cN2 3; cNx 5
Post-treatment T stage, n	ypT0 3; ypTis 2; ypT1 1; ypT2 9; ypT3 9; ypT4 3
Nodal status, n	ypN0 20; node-positive 7 (26%)
Tumor grade, n	G1/low 4; G2 10; G3 1; mucinous 2; no residual invasive tumor 5; not recorded 5
Margin status (recorded in 21)	R0 19; R1 2 (involved circumferential margin)
Lymph nodes harvested, median (range)	10.5 (1–26); n = 18
ICG-FA performed, n (%)	22 (81.5)

One case (a laterally spreading rectal tumor) did not receive neoadjuvant therapy and is staged pTis. Several oncological fields were not uniformly recorded and are reported for available cases only.

### Surgical strategy

**Figure 2:**
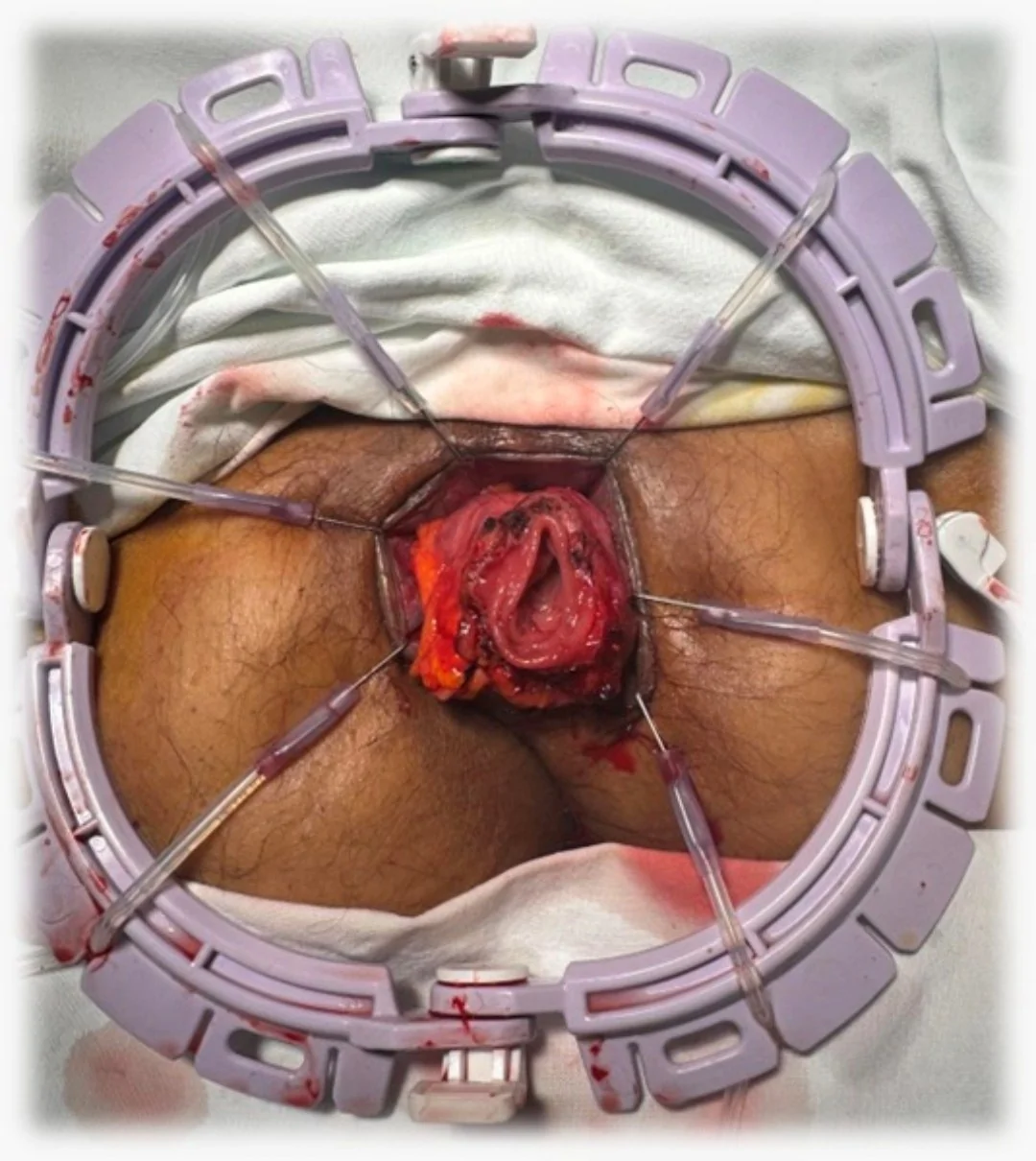
Perineal view through a circular self-retaining retractor showing the completed hand-sewn coloanal anastomosis after intersphincteric resection.

Of the 70 patients with low rectal cancer, 27 underwent sphincter-saving procedures and constitute the focus of this analysis. The remaining 43 patients were treated by APR or other approaches according to the multidisciplinary tumor board recommendation. Within the sphincter-saving subgroup, three operative strategies were used, classified by the resection/dissection plane rather than by the anastomosis:

- Intersphincteric resection (ISR) with hand-sewn coloanal anastomosis and protective loop ileostomy was performed in 7 patients with very low tumors, requiring partial or subtotal removal of the internal anal sphincter to achieve a safe distal margin.
- Ultralow anterior resection with an immediate low colorectal or coloanal anastomosis and protective loop ileostomy was performed in 4 patients, after laparoscopic or open low anterior resection with TME, using either a stapled or a hand-sewn technique depending on the height of the residual rectal cuff (**Figure 2**).
- A two-staged coloanal anastomosis using the Turnbull–Cutait pull-through technique was performed in 16 patients, in whom a temporary stoma was considered undesirable or technically unfavorable, or in whom the proximal colonic length and quality made delayed anastomosis the safer option. In these patients, after TME the proximal colon was exteriorized through the anal canal and resected six to ten days later, when a hand-sewn coloanal anastomosis was constructed without the need for a diverting stoma.

All operations were performed by the same senior surgical team. TME was completed in standard fashion, with high ligation of the inferior mesenteric artery, full splenic flexure mobilization whenever required, and preservation of the autonomic pelvic plexus. The main features of the three techniques used in the sphincter-saving subgroup are summarized in **Table 2**.

**Table 2:** Comparative features of the three sphincter-saving techniques used in our cohort (n = 27).

Feature	Intersphincteric resection (ISR)	Ultralow anterior resection — immediate low colorectal or coloanal anastomosis	Two-staged Turnbull–Cutait pull-through
Patients, n (%)	7 (25.9%)	4 (14.8%)	16 (59.3%)
Typical indication	Very low tumors requiring partial/subtotal internal sphincter excision for a safe distal margin	Low rectal tumors with a sufficient distal cuff after TME for a stapled or hand-sewn anastomosis	Cases in which a diverting stoma is undesirable or technically unfavorable
Anastomotic technique	Hand-sewn coloanal	Stapled or hand-sewn low colorectal/coloanal	Delayed hand-sewn coloanal (day 6–10)
Diverting ileostomy	Yes	Yes	No
ICG-FA performed	Routinely	Routinely	Routinely, prior to colonic exteriorization
Main perioperative concerns	Stump ischemia, anastomotic leak, late stenosis, continence impairment	Anastomotic leak, presacral abscess, stoma-related morbidity	Mucosal necrosis of exteriorized colon, retraction, anastomotic stenosis
Need for second operation	Yes — ileostomy reversal	Yes — ileostomy reversal	Yes — delayed anastomosis (planned, no stoma)

### ICG fluorescence angiography protocol

**Figure 3:**
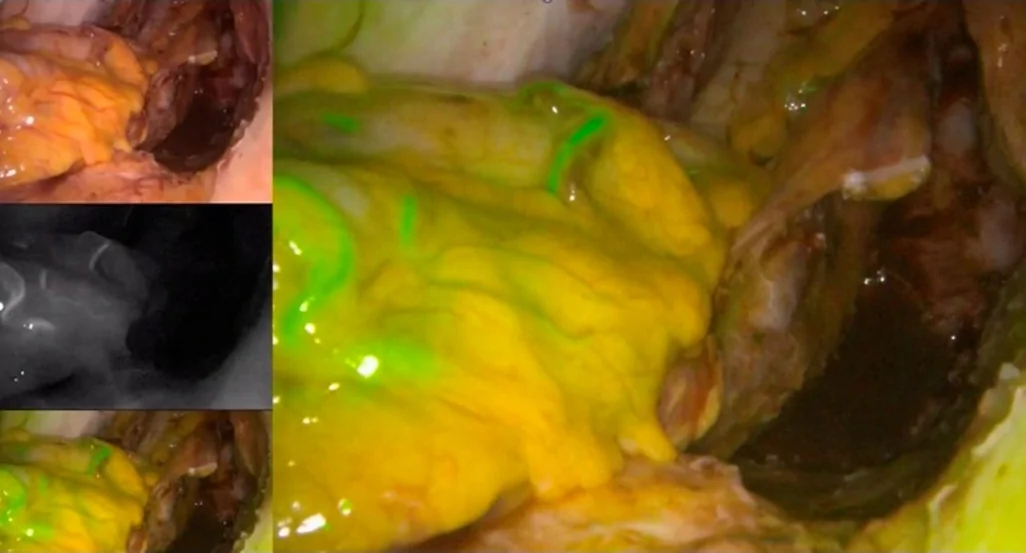
Intraoperative indocyanine green fluorescence angiography of the colonic stump prior to anastomosis: white-light image, near-infrared signal, and color-overlay mode, showing homogeneous and prompt fluorescence at the planned transection line.

In 22 of the 27 sphincter-saving cases (81.5%), intraoperative ICG-FA was used to evaluate the perfusion of the colonic stump. After completion of vascular ligation, mobilization, and selection of the planned transection line, 5–7.5 mg of indocyanine green were injected as an intravenous bolus, followed by a saline flush. Fluorescence visualization was carried out using a dedicated near-infrared imaging system. The cut edge of the colon was considered well perfused when a homogeneous and prompt fluorescent signal was observed within 30–60 seconds of injection. Whenever the chosen segment showed delayed, patchy, or absent fluorescence, the transection line was shifted proximally to a clearly perfused area before construction of the anastomosis. The decision to modify the resection line was always shared between two senior surgeons. A representative composite view is shown in **Figure 3**.

### Postoperative follow-up and outcomes

All patients were monitored for early complications during their hospital stay and at 30, 60, and 90 days after surgery, with subsequent oncological follow-up at three- to six-month intervals. The primary endpoint was the proportion of ICG-FA assessments that prompted a change in the planned proximal transection line, defined as any repositioning of the transection point after fluorescence assessment relative to the line selected by white-light criteria. Secondary outcomes were the overall and specific rate of postoperative complications (stump ischemia, anastomotic leak, anastomotic stenosis, presacral or perianastomotic abscess, and stoma-related complications) and 30-day mortality. Complications were graded according to the Clavien–Dindo classification.

### Statistical analysis

This was a descriptive study, and the analysis was planned accordingly. Continuous variables are reported as median (range) and categorical variables as absolute numbers and percentages. The primary endpoint is reported as a percentage with the corresponding count. Postoperative complications are presented both by type and by Clavien–Dindo grade. Exploratory comparisons across the three operative techniques (intersphincteric resection, immediate low colorectal or coloanal anastomosis, and two-staged Turnbull–Cutait pull-through) used the Fisher exact test for categorical variables and the Kruskal–Wallis test for continuous variables; given the small subgroups, these comparisons were not corrected for multiple testing and are regarded as hypothesis-generating. The study was not powered to detect differences in complication rates, and no formal sample-size calculation was performed. Missing data were not imputed, and denominators are specified where they vary. The per-patient modification status of the resection line was not recorded prospectively, only the aggregate rate; consequently no patient-level comparison between modified and non-modified cases was possible. Analyses were performed using JASP (version 0.19).

## Results and discussion

### Cohort characteristics and operative distribution

Between January 2021 and December 2025, more than 400 patients underwent surgery for colorectal cancer in our department. Seventy of them (17.4%) had low rectal cancer; 27 of these (38.6%) were considered suitable for sphincter-saving surgery, while the remaining 43 patients (61.4%) were managed by APR or alternative non-restorative procedures. The high proportion of APR mirrors the strict oncological selection applied in our institution: when the tumor unequivocally infiltrated the sphincter complex or when patient-related factors made a low anastomosis hazardous, sphincter preservation was deliberately foregone.

Within the sphincter-saving group, intersphincteric resection was performed in 7 patients (25.9%), ultralow anterior resection with an immediate low colorectal or coloanal anastomosis in 4 (14.8%), and two-staged Turnbull–Cutait pull-through coloanal anastomosis in 16 (59.3%). The annual distribution of sphincter-saving procedures over the study period was 4 cases in 2021, 4 in 2022, 5 in 2023, 7 in 2024, and 7 in 2025, reflecting a progressive consolidation of the technique within our institutional practice. The relatively high proportion of two-staged pull-through procedures reflects an institutional preference for avoiding a diverting stoma in selected ultra-low anastomoses.

### Baseline oncological characteristics

The baseline clinicopathological characteristics of the sphincter-saving cohort are summarized in **Table 1**. The median age was 65 years (range 36–85), with 16 men and 11 women; the median tumor distance from the anal verge was 5 cm (range 1–7), and 26 of the 27 patients (96%) received neoadjuvant therapy — only one patient, with a laterally spreading tumor, was managed without neoadjuvant treatment. Clinical stage was recorded in 16 patients and ranged from cT1 to cT4 (cT1 1, cT2 5, cT3 7, cT4 3), with clinically node-positive disease (cN1–N2) in 7. Post-treatment pathology demonstrated a wide spectrum of response and stage: no residual invasive carcinoma in 5 patients (including two early lesions), through ypT1–ypT2 in 10 and ypT3 in 9, to three locally advanced ypT4 tumors. Seven of the 27 patients (26%) were node-positive. A radical (R0) resection was achieved in 19 of the 21 patients in whom margin status was recorded; two patients had an involved circumferential margin (R1) — one with a positive circumferential resection margin on the specimen (ypT3 N1) and one with mesorectal-fascia involvement (ypT4 N1) — both in advanced, node-positive tumors. The median lymph-node yield was 10.5 (range 1–26). These data are reported for the cases in which each variable was available; circumferential margin, distal margin, and nodal yield were not recorded uniformly across the cohort and are presented without imputation.

### Intraoperative impact of ICG fluorescence angiography

In 22 of the 27 sphincter-saving operations, ICG-FA was performed at the moment of choosing the proximal transection line. The technique proved easy to integrate into the operative workflow, added no clinically relevant prolongation of the procedure, and was free of adverse reactions to the dye in our experience. In 9 of the 22 cases (40%; exact binomial 95% CI 21–64%), the planned resection line was modified after ICG assessment: in all but one of these patients, the transection point was moved proximally because the originally chosen segment showed delayed or heterogeneous fluorescence, suggesting marginal perfusion of the cut edge. In one patient, ICG-FA reassured the surgical team about a segment that on macroscopic inspection had appeared dusky, allowing the originally chosen line to be safely maintained. In every case, the modification remained within an unchanged sphincter-preserving plan; ICG-FA did not alter the decision between sphincter preservation and APR in any patient.

**Figure 4:**
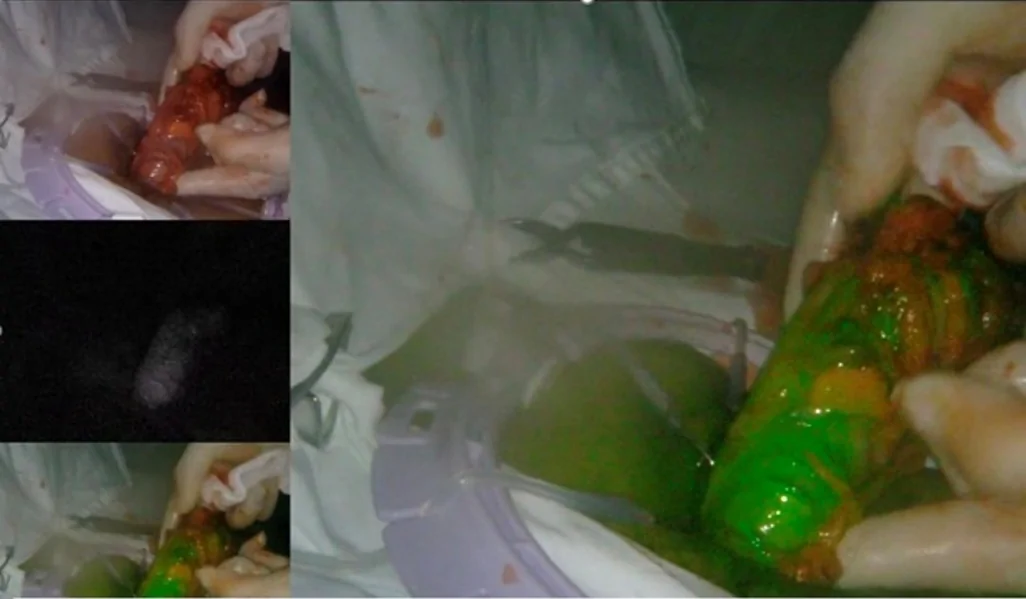
Intraoperative ICG fluorescence assessment of the colonic stump during a two-staged Turnbull–Cutait pull-through procedure; uniform fluorescence of the exteriorized colonic segment confirms adequate perfusion at the planned delayed coloanal anastomosis.

This 40% rate of intraoperative change is substantially higher than figures reported in unselected colorectal series, where modification of the transection line ranges typically between 3% and 20% (7, 9). Our higher proportion is explained by the deliberately challenging case-mix: only patients with very low tumors and high-tension, ultra-low anastomoses were included, in whom the proximal colonic segment had to reach deep into the pelvis after extensive mobilization. In such anatomical conditions, even moderate marginal hypoperfusion can translate into clinically relevant ischemia at the anastomotic line. The ICG signal allowed us to objectivate this hypoperfusion before, rather than after, the construction of the anastomosis. **Figure 4** illustrates a representative perfusion check during a two-staged Turnbull–Cutait pull-through procedure.

### Postoperative morbidity in the sphincter-saving subgroup

Postoperative complications occurred in 15 of the 27 patients (56%); the distribution by Clavien–Dindo grade is shown in **Table 3**. The majority were grade I–II and managed conservatively (transient cytolysis, anemia or electrolyte disturbance, postoperative ileus, a rectovaginal fistula managed conservatively). Major complications (grade ≥IIIb) occurred in a minority and were predominantly anastomotic, including two cases that required takedown of the coloanal anastomosis; one patient developed a critical-care–level complication (grade IV). There was no 30-day mortality. One patient died in hospital more than 30 days after surgery from a myocardial infarction judged independent of the surgical procedure (Clavien grade V); the perioperative (30-day) mortality therefore remained nil. When examined by operative technique, complications occurred in 4 of 7 intersphincteric resections (57%), 2 of 4 immediate low colorectal or coloanal anastomoses (50%), and 9 of 16 two-staged pull-through procedures (56%) (**Table 4**); major complications (Clavien ≥IIIb) were most frequent, in absolute terms, after the pull-through procedure (6 of 16), which also accounted for both anastomotic takedowns and the single grade IV event.

**Table 3:** Postoperative complications by Clavien–Dindo grade (n = 27).

Clavien–Dindo grade	n (%)
Grade I	1 (4)
Grade II	6 (22)
Grade IIIa	0
Grade IIIb	6 (22)
Grade IV	1 (4)
Grade V (in-hospital death)	1 (4)
Overall morbidity	15 (56)
30-day mortality	0 (0)

By type, complications comprised anastomotic leak/dehiscence in 4 patients (2 requiring anastomotic takedown), one rectovaginal fistula managed conservatively, postoperative ileus, and medical complications; one late mucosal prolapse required reintervention. The grade V was a late (>30-day) in-hospital death from an independent myocardial infarction.

**Table 4:** Postoperative complications by operative technique (n = 27).

Technique	Patients, n	Complicated, n (%)	Major (Clavien ≥IIIb), n
Intersphincteric resection	7	4 (57)	1
Immediate low colorectal/coloanal anastomosis	4	2 (50)	1
Two-staged Turnbull–Cutait pull-through	16	9 (56)	6
Total	**27**	**15 (56)**	**8**

Subgroups are small; between-technique comparisons are exploratory. Both anastomotic takedowns and the single Clavien IV event occurred in the pull-through group.

Bold typeset of numbers is only for highlighting purposes.

Overall morbidity was broadly similar across the three approaches (50–57%), and with subgroups of this size no technique showed a statistically meaningful advantage; these comparisons are exploratory and hypothesis-generating. Morbidity after intersphincteric resection is consistent with the published range of 7.7–41% (10, 11). Notably, the two anastomotic takedowns and the single critical-care–level event all occurred in the two-staged Turnbull–Cutait group, so that major complications clustered, in absolute terms, in this (largest) subgroup — a more cautious reading than an unqualified claim of a more favorable septic profile, although the pull-through approach did avoid a diverting stoma in selected patients, consistent with the TURNBULL-BCN randomized trial and recent meta-analyses (12–14).

The oncological profile of the series merits explicit comment. While a radical resection was achieved in the large majority, two patients had an involved circumferential margin and a further three had locally advanced ypT4 disease; these were the tumors in which sphincter preservation was oncologically most debatable, and they underscore that case selection — not technique alone — governs the safety of a restorative approach in the lowest rectum. Conversely, five patients had no residual invasive carcinoma, reflecting favorable downstaging in a neoadjuvant-treated population and, in two instances, early lesions included as part of consecutive practice.

### Clinical relevance, limitations, and conclusions

Two practical lessons emerged from our series. First, sphincter preservation in low rectal cancer should not be presented to patients as a simple alternative to APR: the avoidance of a permanent stoma is a legitimate goal, but the trade-off includes a substantial risk of perioperative morbidity, a diverting ileostomy in most patients, and a non-negligible incidence of low anterior resection syndrome (1, 2, 4, 15). Honest preoperative counselling is essential for shared decision-making. Second, ICG-FA is a particularly useful adjunct precisely in the high-risk subset selected for ultra-low sphincter-saving surgery, where the perfusional margin is most precarious (6–9). It nevertheless remains a largely qualitative test dependent on visual interpretation, with recognized inter-observer variability (9); we mitigated this by requiring a joint judgement by two senior surgeons, and we regard machine-assisted quantification as the natural next step.

The randomized evidence is itself evolving and should temper strong claims. Recent meta-analyses restricted to randomized trials show a significant reduction in clinical anastomotic leak with ICG-FA (pooled odds ratio approximately 0.66–0.69, a relative reduction of roughly one third), with the benefit most pronounced in left-sided and low anterior resections (6, 16). The IntAct multicenter randomized trial, however, did not meet its primary endpoint: clinical (grade B or C) anastomotic leak within 90 days was not significantly reduced, grade C leak rates were comparable between arms, and only a non-significant signal toward fewer clinical leaks was observed (9). IntAct enrolled a broad rectal-cancer population (tumors up to 15 cm from the anal verge undergoing high or low anterior resection) and excluded the extended and ultra-low restorative procedures that define our series; its neutral primary result therefore cannot be extrapolated directly to the ultra-low, high-tension anastomoses in which we observed the greatest intraoperative impact of ICG-FA. Our data should be read in this light: ICG-FA is a valuable and increasingly adopted adjunct that reorients intraoperative decision-making, but whether it reduces anastomotic leak in this specific high-risk subset remains to be demonstrated in controlled studies (17, 18).

This study has several limitations. It is a single-center, observational, descriptive analysis, with a relatively small sphincter-saving cohort and no non-ICG comparator; the absence of a control group precludes any causal inference regarding the effect of ICG-FA on complication rates. The per-patient resection-line modification status was not recorded, only the aggregate rate, and the magnitude of proximal displacement was not captured; these data could not therefore be analyzed at the patient level. Nevertheless, the prospective design, the homogeneity of the surgical team, the standardized indication, and the comprehensive follow-up provide a coherent picture of contemporary practice in a European academic center.

## Conclusions

In a five-year prospective experience involving more than 400 patients with colorectal cancer, of whom 70 had low rectal tumors and 27 underwent sphincter-saving procedures (19), sphincter preservation was feasible and oncologically sound in carefully selected patients but was far from a complication-free alternative to APR: postoperative morbidity reached 56%, with no 30-day mortality. ICG-FA proved a safe, reproducible adjunct that prompted a change of the planned resection line in 40% of the patients in whom it was performed, identifying hypoperfused colonic segments that had appeared macroscopically acceptable, while never altering the sphincter-preservation-versus-APR decision.

In our view, systematic ICG perfusion assessment, combined with rigorous patient selection, multidisciplinary decision-making, and transparent counselling, is a valuable, low-risk, and increasingly adopted adjunct to intraoperative decision-making in sphincter-saving surgery for low rectal cancer. Whether it reduces anastomotic complications cannot be determined from this uncontrolled, single-center cohort. Further multicenter prospective studies, ideally randomized and incorporating standardized fluorescence quantification, are needed to define its role and to identify the subgroups that derive the greatest benefit.

## Data Availability

The raw data supporting the conclusions of this article will be made available by the authors, without undue reservation.
