# Correction: Sphincter-saving surgery versus abdominoperineal resection in low rectal cancer: the role of indocyanine green fluorescence angiography in surgical decision-making

**DOI:** 10.3389/fonc.2026.1971421

**Published:** 2026-09-03

**Authors:** Mihaela C. Misca, Sorin V. Petrea, Roxana D. Boanta, Sorin Aldoescu, Eduard Catrina, Mihaela E. Vilcu, V. Grigorean, V. Strambu, Iulian Brezean

**Affiliations:** 1 Dr I Cantacuzino Clinical Hospital and Carol Davila University of Medicine and Pharmacy, Bucharest, Romania; 2 Parhon Endocrinology Institute, Bucharest, Romania; 3 Bagdasar-Arseni Emergency Clinica Hospital Bucharest and Carol Davila University of Medicine and Pharmacy Bucharest, Bucharest, Romania; 4 Dr Carol Davila Nephrology Hospital Bucharest Romania and Carol Davila University of Medicine and Pharmacy Bucharest, Bucharest, Romania

**Keywords:** abdominoperineal resection, coloanal anastomosis, fluorescence angiography, indocyanine green, intersphincteric resection, low rectal cancer, pull-through, sphincter-saving surgery

The **Acknowledgement** section was missing from the article. The correct **Acknowledgement** reads:

“Publication of this paper was supported by the University of Medicine and Pharmacy Carol Davila, through the institutional program Publish not Perish.”

The original version of this article has been updated.

